# Synthesising 30 Years of Mathematical Modelling of *Echinococcus* Transmission

**DOI:** 10.1371/journal.pntd.0002386

**Published:** 2013-08-29

**Authors:** Jo-An M. Atkinson, Gail M. Williams, Laith Yakob, Archie C. A. Clements, Tamsin S. Barnes, Donald P. McManus, Yu Rong Yang, Darren J. Gray

**Affiliations:** 1 School of Population Health, Infectious Disease Epidemiology Unit, University of Queensland, Brisbane, Australia; 2 Queensland Alliance for Agriculture and Food Innovation, The University of Queensland, Gatton, Australia; 3 Molecular Parasitology Laboratory, Queensland Institute of Medical Research, Brisbane, Australia; 4 Griffith Health Institute, Griffith University, Brisbane, Australia; 5 Ningxia Medical University, Yinchuan, Ningxia Hui Autonomous Region, People's Republic of China; WHO/FAO Collaborating Center for Parasitic Zoonoses, Denmark

## Abstract

**Background:**

Echinococcosis is a complex zoonosis that has domestic and sylvatic lifecycles, and a range of different intermediate and definitive host species. The complexities of its transmission and the sparse evidence on the effectiveness of control strategies in diverse settings provide significant challenges for the design of effective public health policy against this disease. Mathematical modelling is a useful tool for simulating control packages under locally specific transmission conditions to inform optimal timing and frequency of phased interventions for cost-effective control of echinococcosis. The aims of this review of 30 years of *Echinococcus* modelling were to discern the epidemiological mechanisms underpinning models of *Echinococcus granulosus* and *E. multilocularis* transmission and to establish the need to include a human transmission component in such models.

**Methodology/Principal Findings:**

A search was conducted of all relevant articles published up until July 2012, identified from the PubMED, Web of Knowledge and Medline databases and review of bibliographies of selected papers. Papers eligible for inclusion were those describing the design of a new model, or modification of an existing mathematical model of *E. granulosus* or *E. multilocularis* transmission. A total of 13 eligible papers were identified, five of which described mathematical models of *E. granulosus* and eight that described *E. multilocularis* transmission. These models varied primarily on the basis of six key mechanisms that all have the capacity to modulate model dynamics, qualitatively affecting projections. These are: 1) the inclusion of a ‘latent’ class and/or time delay from host exposure to infectiousness; 2) an age structure for animal hosts; 3) the presence of density-dependent constraints; 4) accounting for seasonality; 5) stochastic parameters; and 6) inclusion of spatial and risk structures.

**Conclusions/Significance:**

This review discusses the conditions under which these mechanisms may be important for inclusion in models of *Echinococcus* transmission and proposes recommendations for the design of dynamic human models of transmission. Accounting for the dynamic behaviour of the *Echinococcus* parasites in humans will be key to predicting changes in the disease burden over time and to simulate control strategies that optimise public health impact.

## Author Summary

Echinococcosis is a complex zoonosis for which there is sparse evidence on the effectiveness of control strategies in diverse settings. This presents significant challenges for the design of effective public health policy against this disease. Mathematical modelling is a useful tool for simulating control packages under locally specific transmission conditions to inform optimal timing and frequency of phased interventions for cost-effective control of echinococcosis. This systematic review of 30 years of *Echinococcus* modelling discusses the importance of six key epidemiological mechanisms underpinning models of *Echinococcus granulosus* and *E. multilocularis* transmission and establishes the need to include a human transmission component. Accounting for the dynamic behaviour of the *Echinococcus* parasites in humans will be key to predicting changes in the disease burden over time and to simulate control strategies that optimise public health impact.

## Introduction

Echinococcosis is a parasitic disease caused by the larvae of fox and dog cestode worms of the genus *Echinococcus*. It is a complex zoonosis that has domestic and sylvatic lifecycles, and a range of different intermediate and definitive host species. The two most clinically relevant species are *E. granulosus* and *E. multilocularis*, which cause cystic and alveolar echinococcosis respectively. Transmission of both is influenced by climate change and anthropogenic environmental factors, mediated by changes in animal population dynamics, spatial overlap of competent hosts and the creation of favourable weather conditions for egg survival 1–4. Humans are incidental hosts and, in most cases, do not contribute to continuance of the parasite life cycle, except under unique circumstances [5]. However, they bear the burden of serious morbidity and mortality as well as social and economic consequences [6]–[8]. There is an effective vaccine for use in sheep against *E. granulosus* [9], but there is currently no human vaccine, and the disease is not readily detected until it is at an advanced stage without expensive public health screening comprising imaging studies (e.g. ultrasound) [10].

There is a lack of evidence for effective and sustainable control strategies for *E. granulosus* or *E. multilocularis* across regions that vary in endemicity and transmission conditions. Lessons learned from previous infectious disease elimination campaigns indicate that complex diseases cannot be successfully eliminated using a one-size-fits-all approach, but rather, that control strategies should be tailored to local contexts [11], [12]. The complexities of echinococcosis, the diverse environmental conditions that support its transmission, and the sparse evidence on the effectiveness of control strategies in diverse settings, provide significant challenges for policy makers attempting to make informed control decisions.

Such issues have given rise to the popularity of mathematical modelling to simulate control packages under locally specific transmission conditions. Importantly, modelling negates the expense of trialling scenarios in the field and provides evidence for optimal timing and frequency of phased control interventions. Model output can also be integrated with economic analyses to determine and compare the cost-effectiveness of different control and elimination interventions, alone and as part of an integrated approach.

Early models of *E. granulosus* and *E. multilocularis* [13], [14] described the basics of transmission and these have since been adapted based on advances in epidemiological understanding arising from field data from Australia, New Zealand, Europe, the Middle East and central Asia [15]. Models can vary from simple representations of the system to detailed epidemiological frameworks with large numbers of parameters [16]. To date, *Echinococcus* transmission models have focussed primarily on the life cycle in animal definitive and intermediate hosts and have not included the transmission pathway to humans. Although humans rarely contribute to transmission [5] they are indeed a host and valuable insight into the impact of interventions targeting both definitive and intermediate hosts can be gained by their inclusion into *Echinococcus* transmission models. While the risk of echinococcosis in humans and the impact of control interventions (targeting definitive hosts) on this risk have indeed been discussed in a number of papers detailing animal models of *E. multilocularis* – it is noteworthy that this has not been done for *E. granulosus* – this risk is based on the assumption that the number of human cases is proportional to the quantity of parasite eggs deposited in the environment [17]–[19]. The assumption that human risk increases linearly with increased prevalence of infected foxes is acknowledged to be an over simplification [18], although this is still an important indicator of risk. These *E. multilocularis* risk models also do not account for heterogeneous human exposure arising from varying spatial overlap of hosts, or socioeconomic and environmental conditions affecting subpopulations of humans in endemic areas. Furthermore, they are unable to simulate preventive interventions targeting humans and hence the impact of these on infection and subsequent morbidity and mortality.

Developing echinococcosis transmission models incorporating both animal and human hosts will be important for exploring the dynamics of transmission to humans [20], for predicting changes in the human disease burden over time, and will be essential for public health planning of control strategies. Much progress has been made over the last 30 years in modelling the lifecycle of *Echinococcus spp.* in animal hosts. The aims of this review were to discern the epidemiological mechanisms underpinning models of *E. granulosus* and *E. multilocularis* transmission and to propose recommendations for the future design of dynamic models of *E. granulosus* and *E. multilocularis* transmission that incorporate the human host.

## Methods

### Search strategy

A search was conducted of all relevant articles published up until July 2012, identified from the PubMED and Web of Knowledge databases. Key terms used in the search strategy included: ‘mathematical model OR models OR computer model OR decision support system OR decision tree’ AND ‘echinococcus OR echinococcosis OR *E. granulosus* OR *E. multilocularis*.’ The search was limited to English language publications. Review of bibliographies of papers was also carried out to ensure completeness of inclusion of all relevant mathematical models.

### Study selection

**Figure 1:**
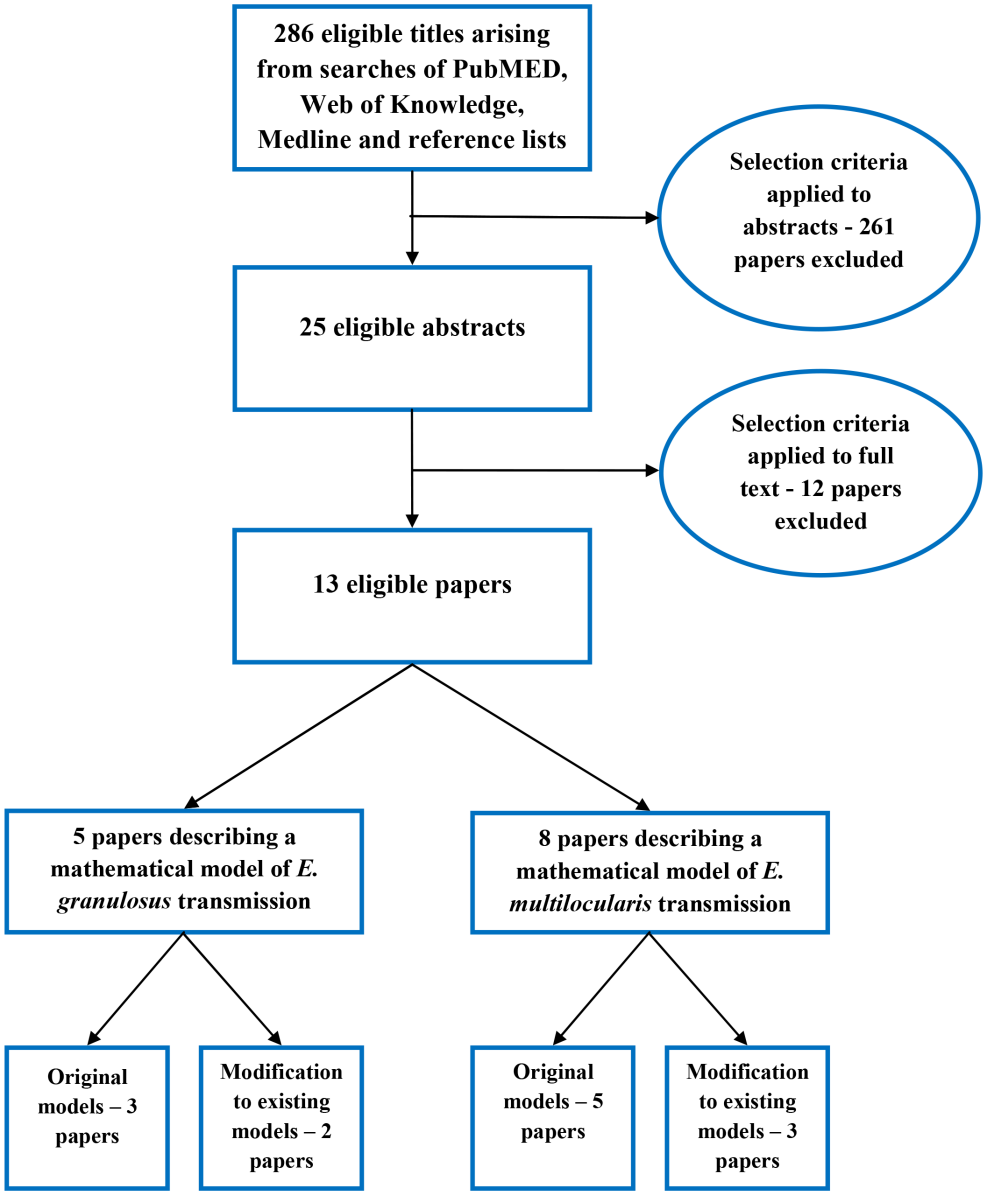
Flow chart of paper selection process to identify relevant mathematical models.

Papers eligible for inclusion were those describing the design of a new model, or modification of an existing mathematical model of *E. granulosus* or *E. multilocularis* transmission. Papers were excluded if they described: statistical risk modelling rather than dynamic, mechanistic modelling of *Echinococcus spp.* lifecycles; processes at a microbiological level with focus on an individual component of the life cycle; generic mathematical models of parasitic disease transmission; or if they described the implementation of an existing model without recommendations for modification of the model. In addition, review papers of models described elsewhere were excluded. The process of study selection is summarised in Figure 1. Appendices S1 and S2 provide summaries of *E. granulosus* and *E. multilocularis* models included in this review and their specific assumptions.

## Results and Discussion

A total of 13 eligible papers were identified, five of which described mathematical models of *E. granulosus* and eight that described *E. multilocularis* transmission. These are predominantly population-based compartmental models although some authors have explored individual-level transmission dynamics. The majority of models identified were fitted to field data on disease prevalence in host species. However, two *E. multilocularis* models [21], [22] focussed on parasite biomass (i.e. compartments of the model represent eggs in the environment, protoscoleces in small mammals, and worms in foxes) rather than the infection status of host populations. This was reported to be valuable for exploring the components of the life-cycle of *E. multilocularis* that occur in the environment, as well as in definitive and intermediate hosts [22].

While the modelling of parasite biomass has not been carried out for *E. granulosus* to date, this may be relevant given the possibility that more than one dog may feed on an infected sheep and the probability of each becoming infected will be influenced not only by the number of cysts but by the number of protoscoleces in each cyst. In modelling the dynamics of the egg, larvae, and adult worm stages of *Echinococcus spp.* (i.e. parasite biomass) in hosts and the environment, the reproductive number derived is different in interpretation than models focussing on infection status of host populations, in that it reflects the expected number of mature parasites produced during the life-span of a single parasite rather than the average number of secondary infections arising from a single infected host [22].

The models included in this review varied primarily on the basis of six key features that were differentially incorporated in their design. These are: 1) the inclusion of a ‘latent’ class (with time delay from host exposure to infectiousness); 2) an age structure for definitive and/or intermediate hosts; 3) the presence of density-dependent constraints; 4) accounting for seasonality; 5) stochastic parameters; and 6) inclusion of a spatial and risk structures. The conditions under which these mechanisms may be important for inclusion in models of *Echinococcus* transmission are discussed. Table 1 also summarizes the inclusion of these key elements in each of the models.

**Table 1:** A summary of the presence of key elements in each of the *E. granulosus* and *E. multilocularis* transmission models included in this review.

Model name	Model type§	Inclusion of a ‘latent’ class*	Age Structure	Density dependent mechanisms	Seasonality	Stochastic parameters	Spatial/risk structure
**Mathematical models of ** ***E. granulosus***							
**Roberts model** [14]	**Compartmental**		**✓**				
**Modified Roberts model (a)** [32]	**Compartmental**		**✓**	**✓**		**✓**	
**Modified Roberts model (b)** [31]	**Compartmental**		**✓**	**✓**			
**Torgerson model** [30]	**Compartmental Prevalence- based model**		**✓**	**✓**		**✓**	
**Heinzmann model** [29]	**Simulation**		**✓**		**✓**		**✓**
**Mathematical models of ** ***E. multilocularis***							
**Roberts model** [13]	**Compartmental**	**✓**					
**Modified Roberts model** [28]	**Compartmental**						**✓**
**Ishikawa model** [17]	**Compartmental**	**✓**	**✓**		**✓**	**✓**	
**Modified Ishikawa mixed model** [27]	**Compartmental/Simulation**	**✓**	**✓**	**✓**	**✓**	**✓**	
**Hansen ‘Echi’ model** [26]	**Simulation**	**✓**		**✓**		**✓**	**✓**
**Takumi model** [22]	**Compartmental**			**✓**			
**Modified Takumi model** [21]	**Compartmental**						**✓**
**Kato model** [18]	**Compartmental**			**✓**			

* The inclusion of an exposed but not yet infectious class and time delay.

§ Compartmental models – ‘individuals in the population are subdivided into broad subgroups and the model tracks the infection process for these individuals collectively;’ Simulation model – ‘model tracks the infection process for every individual in the population’ [10].

### Inclusion of a ‘latent’ class and delays

Maturation of *E. granulosus* and *E. multilocularis* worms in the definitive host is thought to take approximately 6 weeks [8]. Maturation of cysts in intermediate hosts can differ not only between the two species, but also between different intermediate host species, particularly for *E. granulosus*. For example, maturation time for *E. multilocularis* cysts in small mammals is estimated at 2–4 months, while for *E. granulosus* cyst maturation can take 8–9 months in wallabies but 2–6+ years in sheep [8], [23], [24]. Time delays in parasite lifecycles tend to attenuate transmission potential because they allow for the possibility of host death between infection and infectiousness [25]. Time delays for parasite maturation are usually incorporated into compartmental models by the inclusion of a ‘latent’ class (i.e. an exposed but not yet infectious class, also referred to as an ‘E’ class). This ‘latent’ class was present in four of the eight *E. multilocularis* models for both definitive and intermediate hosts [13], [18], [26], [27]. Inclusion of a ‘latent’ class, however, does not always contribute qualitatively to the dynamics of a model [25]. For example, a study that resulted in the modification of the original *E. multilocularis* model of Roberts and Aubert (1995) found that exclusion of the ‘E’ class did not alter their conclusions and hence it was omitted and a simpler Susceptible – Infectious (S-I) model used [28]. Therefore, inclusion of a latent class may be more relevant for *E. granulosus* models, particularly those involving the intermediate sheep host where it takes years to reach cyst maturity and hence infectiousness. As such, the importance of the inclusion of a ‘latent’ class is dependent on the life expectancies of the hosts relative to the latent period [25]. Not including the appropriate time delay in the ‘latent’ period when it is warranted (e.g. time to cyst maturation in sheep) could result in an over-estimation of the proportion of infectious hosts in the natural system at any given time [25]. This would lead to inaccurate predictions of the impact of control measures or a failure to accurately estimate the time to disease elimination when simulating control strategies.

### Age structure

Age stratification of hosts was incorporated into the design of the very first *E. granulosus* model [14] and remained an important component of all subsequent models. The intermediate host is universally assumed to remain infected for life and, in the absence of acquired immunity, subsequent exposure to parasite eggs results in the accumulation of cysts in the host, producing a linear relationship between age and the numbers of hydatid cysts [14], [29]–[32].

While the inclusion of an age structure might be assumed to be less relevant for short-lived intermediate hosts of *E. multilocularis* (e.g. the average lifespan of a vole is 7–8 months [33]), in reality, the maturation of cysts occurs relatively quickly (2–4 months) compared with *E. granulosus* (where growth of cysts is slow and variable) [8]. Once an *E. multilocularis* cyst is established, a small mammal such as a vole, remains infected for life, and hence subsequent infections accumulate with increasing age [34]. Evidence of this was found in *Arvicola terrestris* in Switzerland, where increasing prevalence of *E. multilocularis* was observed over several age classes of voles trapped during the study period [34]. Therefore, the age structure of voles and other small mammals may be an important element for inclusion in models of *E. multilocularis*. However, including an intermediate host age structure in *E. multilocularis* models may mask detection of seasonal variation in infection pressure as the age distribution of small mammals can vary considerably between seasons [34]. The use of absolute age estimates has been suggested as a method for overcoming this limitation [34]. This involves determining the date of birth of each small mammal based on its age and trapping day, which is then used to assign mean day temperatures and precipitation (which influence egg survival in the external environment) to each day of life for each animal and to simulate seasonal variation in infection pressure [34].

Age stratification in the definitive host population has also been a characteristic of some *E. granulosus* models [31], [32]. Age-related differences in parasite intensity or prevalence in naturally infected populations of dogs have been reported and are suggested to be related to the acquisition of temporary immunity (discussed in the following section) rather than to any age-related difference in infection pressure [31], [32]. Age stratification of the definitive host is thought to be particularly important when there is likely to be a high turnover in the dog population as this will result in increases in the numbers of younger, more susceptible dogs which may increase infection pressure on human hosts [31]. However, this is dependent on the level of endemicity as classic age-prevalence curves of *E. granulosus* indicate that very young dogs may not survive long enough to become infectious [35]. The inclusion of an age structure in the definitive (fox) host when modelling *E. multilocularis* occurred as a result of field data showing higher worm burdens in juvenile foxes compared with adult foxes in Hokkaido, Japan [27] and is also thought to allow the model to more realistically reflect population dynamics by assigning different death rates to hosts of varying age [17], [27].

### Density-dependence mechanisms

Density-dependent constraints are factors that regulate population growth [36], and have been shown to be critical in simulating the population biology and control of parasites [37]. The absence of expression of density-dependent constraints in a mathematical model of *Echinococcus spp.* makes elimination of parasite species theoretically easy. However, it has been acknowledged that this may not be the case in a natural setting [13], [20], [38]. In the models included in this review, the density-dependent constraints discussed are related to host demography (i.e. the population density of definitive and intermediate hosts) and natural immunity (which regulates parasite abundance). Decisions regarding the inclusion or exclusion of such structural assumptions may have a marked effect on disease projections and the impact and cost-effectiveness of control strategies [39].

#### Demography

Very few models included in this review incorporated the effects of fluctuations in population density of definitive or intermediate hosts on transmission dynamics. We assume a constant population size is valid for short duration diseases that have limited effects on host mortality [40]. However, for endemic diseases present in populations that change substantially, there is a complex relationship between population demographics and disease dynamics that can have important epidemiological effects that should not be ignored [40]. In all models of *E. granulosus*, transmission is assumed to take place in a closed community (with deaths of hosts replaced with susceptible newborns). This may be a reasonable assumption for regions where dog and sheep populations are relatively stable. However, future models applied to developing country contexts may need to consider the effect of the rapidly increasing demand for livestock products resulting in expansion of livestock industries and investment in more efficient slaughtering infrastructure [41], [42].

For *E. multilocularis* models, sylvatic host populations that would be expected to fluctuate seasonally have the potential to significantly influence transmission intensity. Two *E. multilocularis* models from Japan therefore accounted for seasonally dynamic host populations because the primary definitive host, the red fox (*Vulpes vulpes*), and intermediate host, the grey-sided vole (*Clethrionomys rufocanus*), showed marked seasonal variations in population size [17], [27]. However, it is argued by others that introducing seasonally dynamic host populations would add unnecessary complexity and provide results that are unlikely to be quantitatively influenced [13], particularly if the overall annual growth rate of host populations is negligible. Other influences on host dynamics that have been identified as potentially important for inclusion in *E. multilocularis* models are contexts where there are 1) higher rates of death of juvenile foxes; 2) definitive host migration (such as in the arctic fox of the tundra zone of Eurasia and North America); and 3) large scale small mammal population variations due to changes in habitat composition (e.g. resulting from anthropogenic environmental influences such as deforestation and overgrazing) [17], [27], [43].

In the two models that accounted for variation in host population densities, fluctuations resulted from age- and season-dependent variations in birth and death rates, and annual growth rates of both definitive and intermediate hosts were assumed to be stable [17], [27]. A reported disadvantage of models assuming annual growth proportional to population size is that they fail to account for finite resources that eventually limit growth [44]. It has therefore been suggested that, to account for the carrying capacity of the local environment, density- dependent restrictions should be placed on population growth if it is to be included in dynamic transmission models [44]. While not having yet been applied to *Echinococcus spp.* models, accounting for density-dependent population growth rates of intermediate and definitive hosts would be most relevant to the sylvatic cycle of *E. multilocularis*. This could be achieved with the simple inclusion of logistic population growth. Alternatively, maintaining the assumption of constant rodent population density could be justified by the argument that different species of hosts have asynchronous fluctuation patterns in their densities which roughly provides a stable overall presence of intermediate hosts [28]. Understanding the biodiversity of intermediate hosts of *E. multilocularis* in a specific area as well as their life expectancies will be particularly important before making assumptions about whether or not it will be necessary to account for varying population density in the model [22].

#### Natural immunity

Modelling of *E. granulosus* data to date has consistently suggested a lack of regulation of the parasite population by intermediate host natural immunity [14], [30], and this assumption is consistent across all models included in this review. In contrast, the presence of natural immunity in the definitive host has been debated in the literature. Earlier mathematical models fitted to data from Australia [14] and China [35] assessed the presence (if any) of acquired immunity in the definitive host as having negligible impact on prevalence of *E. granulosus* in these hosts. This conclusion may have been a consequence of insufficient definitive hosts surviving long enough to become infectious and contribute to transmission, low infection pressure in these settings, or the inadequate sampling methods used which failed to capture sufficient numbers of older dogs [14], [32], [35]. In contrast, later models fitted to data from Tunisia [45], Kazakhstan [46], China [47] and Morocco [48] indicated the presence of a density-dependent feedback mechanism in high prevalence areas suggesting that immunity to *E. granulosus* is acquired by definitive hosts. The acquisition of immunity is further supported by experimental data that have shown cellular and humoral immune responses in dogs and resistance to re-infection following multiple exposures and suppression of egg production following single high dose exposure to *E. granulosus* [24], [47], [49]–[51].

A similar mechanism is thought to occur with *E. multilocularis*. Results from fox dissections showed juvenile foxes had a greater abundance of worms than adults and field data from a focal area of high *E. multilocularis* prevalence were found to comply best with models that account for foxes acquiring partial immunity [26], [27]. It is unclear whether control programs that focus on de-worming of foxes (with praziquantel) alter the immune competence of the fox [26]. In addition, the presence of acquired immunity in foxes in high endemic areas and its absence in low endemic areas suggests that attempts at controlling parasite transmission (without achieving elimination) may be attenuated by simultaneous reductions in the development of acquired immunity. Therefore, future modelling of interventions should test the effect of including an endemicity threshold, below which the immunity-related density-dependent feedback mechanism in the definitive host is inactivated. To date this has not been incorporated in mathematical models of *E. granulosus* and *E. multilocularis*.

#### Seasonality

Egg survival time in the environment has been found to impact the duration of control programs required for disease elimination [22], [26]. Seasonal conditions that favour egg survival (namely cool temperature and humidity that characterise winter in central Europe and other endemic regions) may lead to their accumulation in the environment resulting in a higher infection pressure during this period compared with the rest of the year [17], [27], [29], [34]. One *E. granulosus* model and two *E. multilocularis* models addressed the issue of seasonality [29]. Authors modelling *E. granulosus* found that prevalences of the disease in simulations accounting for seasonality, were not dissimilar to those produced without the inclusion of seasonal effects [29]. However, these authors admit that their use of seasonal averages may not be as important as intra-seasonal variations of temperature and precipitation, which were not accounted for in their simulation model, as changes in soil moisture/humidity, direct sunlight and high temperatures are known influences on the number of viable eggs in the environment and hence the infection pressure in susceptible intermediate hosts [29], [52], [53]. Therefore, accounting for intra-seasonal variations in temperature and precipitation may be an important consideration for future models of both *E. granulosus* and *E. multilocularis*.

In addition to their potential influence on egg survival time, seasonal effects on *E. multilocularis* host population behaviour may also influence transmission [17], [27]. There is evidence of seasonal variations in fox predation behaviour with higher predation rates found during autumn when small mammal density is usually higher than in other seasons [34], [54]–[56]. This corroborates the theory of increased accumulation of *E. multilocularis* eggs during the winter months following the 2–3 months of parasite development within infected foxes before they shed eggs into the environment [34]. The lowest level of fox predation is assumed to occur during the winter months when excessive depth of snow limits small mammal availability [17]. Hence, in two *E. multilocularis* models, a feeding habit function (average number of small mammals ingested per day) was introduced which is dependent on snowfall and small mammal density [17], [27]. Therefore, accounting for seasonal mechanisms is reported to be important, particularly for *E. multilocularis* models, as they allow more precise analysis of transmission patterns and are valuable for informing the development of more targeted, cost-effective control strategies [17].

### Stochastic parameters

Accounting for stochasticity in parameter values is particularly important when modelling small populations or low disease prevalence where such an effect could produce local extinction or ‘fadeout’ of a disease [25], [57]. In addition, modelling stochasticity allows predictions to capture variability in the epidemic profile in order to better understand the potential for disease persistence and the likely accuracy of the forecasts made, so as to better inform control and elimination strategies [57]. Two of the five *E. granulosus* models and three of the eight *E. multilocularis* models considered in this review incorporated stochasticity in their parameter values [17], [26], [27], [30], [46]. The authors of these models reported that parameter variability was captured in instances where there was: an absence of evidence for specific parameter values, unexplained variability in parameter values from surveillance data or reported in the scientific literature, and when there was uncertainty regarding the capture rate of intermediate and definitive hosts (i.e. capture rate is calculated using an estimate of the total size of the host population) [17], [30]. In the reviewed *E. multilocularis* models, some specific parameters that were modelled stochastically included: fox population dynamics, worm burden in foxes, average number of eggs excreted per day by infected foxes, number of infected small mammals harbouring fertile cysts, and the basic infectious contact rate [17], [26], [27].

In the *E. granulosus* models, some specific parameters for which values could only be estimated from data or that displayed wide variability included: overall or age stratified infection pressure to both intermediate and definitive hosts, life expectancy of the parasite in dogs, time to maturity of cysts in sheep, age of feeding of sheep to dogs and the acquisition and loss of immunity in dogs [30], [32]. In addition, there can be considerable uncertainty in baseline dog surveillance data obtained to inform parameter values for the definitive host model due to the absence of accurate dog population figures and hence uncertain capture rate of dogs [30]. In such circumstances, Monte-Carlo simulation allows this uncertainty to be quantified by modelling the variability and predicting best- and worst-case scenarios [30].

### Spatial or risk structure

Spatial aggregation and heterogeneous exposure risk are two characteristics of *E. granulosus* and *E. multilocularis* transmission that are not frequently accounted for in the mathematical modelling of echinococcosis. Spatial aggregation can occur as a result of over-dispersion of the parasite in host populations, where a small proportion of animals harbour most of the parasite population, and there is heterogeneous distribution of *Echinococcus* eggs in the environment, both of which influence exposure risk to animal and human hosts [58]. Exposure risk can also be influenced by the spatial overlap of hosts. Explicit inclusion of spatial and contact structures can improve predictions of *Echinococcus* transmission at the population level as well as in the generation of risk mapping in order to target interventions. The inclusion of explicit spatial and contact structures is best achieved by more sophisticated simulation models that are able to assign a constrained set of exposure conditions to each individual in a host population [59]. Explicit inclusion of risk structure has only been partially realised in one of the five *E. granulosus* models, where the authors assigned a random contact rate to each individual sheep at birth and hence the model reflects heterogeneous infection of sheep in the population at any given time [29]. In addition, one of the eight *E. multilocularis* models assigned spatially explicit conditions to each fox in the population and modelled them individually to explore factors that contribute to the heterogeneous distribution of infected foxes and to explain the rapid resurgence of the disease following cessation of control measures [26].

#### Modelling to understand spatial aggregation

A study on the effect of age, spatio-temporal and season-related factors on the prevalence of *E. multilocularis* in Zurich, Switzerland found that transmission is primarily influenced by spatial factors that create micro-foci of high infection pressure [34]. Several hypotheses exist to explain this spatial aggregation. Firstly, it has been suggested that over-dispersion of parasites in the fox population results in a spatially clustered depositing of eggs in faeces within the home range of the small proportion of infected foxes (with scats distributed either homogeneously or heterogeneously within that range) [26], [58]. In addition, spatial clustering may also result from the heterogeneous distribution of small mammal populations or the increased predation by foxes of infected animals because of their reduced mobility (due in part to destruction of liver tissue from expanding cysts) which would result in constant re-infection of foxes occupying that territory [26], [58]. Finally, spatial aggregation may be explained by differential mortality of *Echinococcus* eggs in the environment as a result of landscape characteristics that influence egg survival (e.g. egg survival is generally best in cool, humid areas such as riverbanks). This would result in heterogeneous availability of viable eggs which infect only the subpopulation of small mammals occupying that habitat [26], [58].

There have been some important findings reported from previous spatial models. Using a spatially explicit simulation model of *E. multilocularis*, Hansen *et al.* (2004) suggested that landscape characteristics that differentially influence egg survival lead to heterogeneous availability of infectious eggs and thus a clumped distribution of infected intermediate hosts. This indicates that while seasonality may be an important influence on *E. multilocularis* risk to intermediate hosts (as discussed previously) it does not completely explain the heterogeneity. This *E. multilocularis* model was the first to be rigorously and quantitatively validated across a wide range of parameter variations expected in the natural system to determine the robustness of, and to differentiate between, different model scenarios [58]. Inclusion of a spatial structure in *Echinococcus* modelling has also been useful to demonstrate growth and spatial parasite spread, quantify human risk based on spatial overlap of hosts, and has been found to more closely reproduce surveillance data than non-spatial equivalent models [19], [21]. Despite these valuable insights, the development and practical use of spatially explicit models are still quite nascent. In future, such models may benefit, in the case of *E. multilocularis*, from differentiation between urban and rural foxes given the potential differences in their population density and size of their home ranges [27]. Existing spatially explicit models can be modified to represent real landscapes and be better used to support local-level decision making for control strategies [26]. In addition, when compared with mass screening, spatially explicit modelling offers a cost-efficient method of locating emerging micro-foci of transmission [1].

#### Modelling to understand risk

The mass action principle is a feature of almost all models included in this review. This assumes that there is homogenous mixing of host populations and equal opportunity for each host to come in contact with infectious materials, which may not be an appropriate assumption for accurately modelling *Echinococcus* transmission [26]. Considering the definitive host for *E. granulosus*, human behavioural factors play an important role in the exposure of dogs to infectious material, either through poor dog control and hence increased scavenging behaviour, or by deliberately feeding dogs the offal from infected intermediate hosts [60]. Since human behaviour is influenced by social, cultural and economic factors, accounting for heterogeneous risk in models of *E. granulosus* is potentially important but will be difficult without establishing a mechanism by which human influences on contact patterns between dogs and infected hosts can be simulated robustly. Considering contact risk between the intermediate host and infectious eggs in the environment, data sets from Jordan [61] and Kazakhstan [32] have been used to model the acquisition of *E. granulosus* infection. It was shown that clumped sources of infection (parasite eggs in dog faeces) results in heterogeneity of acquisition by intermediate hosts which is hypothesised to be a result of behavioural differences between pasturing sheep or due to differences in their immune system [62]. In addition, to more accurately reflect heterogeneous risk of infection in the human population one *E. multilocularis* model divided the egg production stage in foxes into two classes according to output; low and high egg producing classes [17]. Accounting for heterogeneity in contact between intermediate hosts and infectious eggs in the environment will be important for modelling the transmission dynamics of both *E. granulosus* and *E. multilocularis* [62].

Given that parasites in general are well known to affect their host's behaviour in order to potentiate transmission, inclusion of heterogeneous contact patterns in the modelling of *E. multilocularis* may be an important consideration [63]–[66]. Of the *E. multilocularis* models surveyed, only one considered whether or not there is increased susceptibility of infected small mammals to predation [28]. Currently there is limited evidence to support this hypothesis but these authors suggested the possibility that reduced mobility of the infected intermediate host arises from rapid proliferation of the metacestode stage resulting in an extended abdomen and thus increasing their vulnerability to predation [28]. In this model, increased susceptibility of infected small mammals to predation was accounted for by increasing the likelihood that individual prey taken by a predator will be infectious [28]. This enhances species resilience and implies that upon cessation of control activities there would be a rapid return to pre-control prevalence levels [26], [28]. Parasite-induced vulnerability to predation of the intermediate host has also been suggested in the *E. granulosus* wolf-moose transmission cycle with the escape behaviour of the moose thought to be modified by the presence of cystic echinococcosis in the lungs [67]. However, little empirical evidence exists to determine the relationship between intermediate host hydatid infection, predation risk and transmission rates.

Over-dispersion of the parasite in both definitive and intermediate hosts was accounted for in almost all models included in this review by modelling aggregation using a negative binomial distribution. However, it has been argued that while the negative binomial function represents a convenient method for fitting highly aggregated abundance data to models of endemic equilibrium, its use in dynamic modelling of parasite control scenarios is inappropriate due to the loss of biological tractability [68]. More recent modelling of *E. granulosus* has shown that a compound mixed Poisson process with a zero-truncated negative binomial distribution provides a more adequate fit for the acquisition of cysts from aggregated infectious material (parasite eggs within dog faeces) and heterogeneous exposure within the pasturing sheep population [29]. In addition, a shot noise process (an extension of the compound Poisson process), which allows death of parasites in a host to be modelled, was found to provide good fit to the aggregated distribution of *E. granulosus* parasites in dogs [29].

### Conclusions and recommendations for future modelling approaches

Empirical evidence for effective and sustainable strategies for the control of *E. granulosus* and *E. multilocularis* transmission is sparse despite the serious health, social and economic consequences of echinococcosis [6]–[8]. The diverse conditions that support transmission provide a challenge for the design of cost effective control strategies across diverse settings. While mathematical models are useful tools in such situations, current *Echinococcus* models do not specifically include the human transmission pathway, nor do they allow for the simulation of interventions (targeting both animal definitive and intermediate hosts and the human host) to assess the impact on human infection. In addition, they do not account for heterogeneous exposure risk in humans that arises from variable spatial overlap of hosts and local environmental conditions that influence transmission. Therefore, in order to design optimal public health strategies to control and eliminate echinococcosis, inclusion of a human transmission component to *E. granulosus* and *E. multilocularis* will be essential. The following recommendations are proposed for modelling transmission in general and for those that also incorporate the human transmission pathway:

1. Deterministic compartmental models are useful for modelling average transmission behaviour in large host populations. Low prevalence of infection (often in small mammal host populations) and complex processes that lead to highly aggregated disease reservoirs and non-random mixing (e.g. heterogeneous contact patterns of susceptible hosts with infectious materials), justify the inclusion of stochastic, individual-level effects in echinococcosis models [39] and this would constitute our recommendation for modelling frameworks of future analyses.
2. Given that both *E. granulosus* and *E. multilocularis* are highly focal in their transmission, coupling of disease mapping with infection dynamics would have great value in developing an understanding of echinococcosis epidemiology. Increased spatial awareness in the transmission of both parasites may improve efforts at targeting infection hotspots in low prevalence contexts, thereby benefiting the cost effectiveness of control.
3. Incorporating a human component will not only serve to improve public health understanding of these two zoonotic diseases, but will also provide a method of ameliorating a key shortcoming described in almost all studies reviewed, namely, the paucity of infection data. As highlighted by this review, the key mechanisms important for inclusion in models of *E. granulosus* and *E. multilocularis* will necessarily be dependent on the context in which the model's use is intended and the local characteristics of the host populations and environmental conditions that are likely to influence transmission. Building complexity into the models should be driven by local context rather than using a standardized approach.

While model complexity does not necessarily equate to realistic predictions, particularly in the absence of reliable parameter data [69], precision in replication of the fundamental natural mechanisms of disease transmission in specific contexts and with the inclusion of transmission to humans, will allow *Echinococcus* spp. models to become useful public health tools for informing the development of targeted, cost-effective control strategies.

## Supporting Information

Appendix S1 **Summary of** ***E. granulosus*** **models included in this review.**
(XLSX)

Appendix S2 **Summary of** ***E. multilocularis*** **models included in this review.**
(XLSX)
